# Diversity and recombination in *Wolbachia* and *Cardinium* from *Bryobia* spider mites

**DOI:** 10.1186/1471-2180-12-S1-S13

**Published:** 2012-01-18

**Authors:** Vera I D Ros, Vicki M Fleming, Edward J Feil, Johannes A J Breeuwer

**Affiliations:** 1 Evolutionary Biology, Institute for Biodiversity and Ecosystem Dynamics, University of Amsterdam, Amsterdam, the Netherlands; 2 Department of Biology and Biochemistry, University of Bath, Bath, UK; 3 Peter Medawar Building for Pathogen Research, Nuffield Department of Clinical Medicine, University of Oxford, Oxford, UK; 4 Current address: Laboratory of Virology, Wageningen University, Wageningen, The Netherlands

## Abstract

**Background:**

*Wolbachia* and *Cardinium* are endosymbiotic bacteria infecting many arthropods and manipulating host reproduction. Although these bacteria are maternally transmitted, incongruencies between phylogenies of host and parasite suggest an additional role for occasional horizontal transmission. Consistent with this view is the strong evidence for recombination in *Wolbachia*, although it is less clear to what extent recombination drives diversification within single host species and genera. Furthermore, little is known concerning the population structures of other insect endosymbionts which co-infect with *Wolbachia*, such as *Cardinium*. Here, we explore *Wolbachia* and *Cardinium* strain diversity within nine spider mite species (Tetranychidae) from 38 populations, and quantify the contribution of recombination compared to point mutation in generating *Wolbachia* diversity.

**Results:**

We found a high level of genetic diversity for *Wolbachia*, with 36 unique strains detected (64 investigated mite individuals). Sequence data from four *Wolbachia* genes suggest that new alleles are 7.5 to 11 times more likely to be generated by recombination than point mutation. Consistent with previous reports on more diverse host samples, our data did not reveal evidence for co-evolution of *Wolbachia* with its host. *Cardinium* was less frequently found in the mites, but also showed a high level of diversity, with eight unique strains detected in 15 individuals on the basis of only two genes. A lack of congruence among host and *Cardinium* phylogenies was observed.

**Conclusions:**

We found a high rate of recombination for *Wolbachia* strains obtained from host species of the spider mite family Tetranychidae, comparable to rates found for horizontally transmitted bacteria. This suggests frequent horizontal transmission of *Wolbachia* and/or frequent horizontal transfer of single genes. Our findings strengthens earlier reports of recombination for *Wolbachia*, and shows that high recombination rates are also present on strains from a restrictive host range. *Cardinium* was found co-infecting several spider mite species, and phylogenetic comparisons suggest also horizontal transmission of *Cardinium* among hosts.

## Background

*Wolbachia* and *Cardinium* are intracellular bacteria infecting a wide range of arthropod species. They have been classified as reproductive parasites, being able to manipulate their host's reproductive system in order to promote their own transmission [1-3]. Recently, beneficial effects of *Wolbachia* have been identified as well, as *Wolbachia* can protect hosts against virus infection [4,5]. *Cardinium* may also exert beneficial effects [6] and in many other cases the effect of *Wolbachia* or *Cardinium* is unknown. *Wolbachia* is well studied and is widespread among arthropods and nematodes. It is estimated that around 66% of all insects are infected with *Wolbachia *[7]. This diverse genus has been subdivided into 11 “supergroups” (A-K) on the basis of molecular phylogenetic analysis [8-13]. *Cardinium* was more recently discovered and has so far been found in 6-7% of all arthropods, though seems to be more common in Chelicerates than in insects [2,14-18]. *Wolbachia* and *Cardinium* have been found co-infecting the same host species [2,15,17-21].

Although *Wolbachia* and *Cardinium* are generally considered to be clonally inherited via vertical transmission, there is now a large body of molecular evidence for discordant phylogenies of host and endosymbiont [22-29]. Distantly related *Wolbachia* or *Cardinium* strains can infect closely related host species, and closely related strains may infect distantly related host species. Such patterns suggest horizontal transmission of bacteria (or at least of some bacterial genes) between hosts, although direct evidence for horizontal transmission is rare [30-32].

Horizontal transfer has been further supported by evidence for recombination [33]. For *Wolbachia*, recombination has been found between genes (intergenic) as well as within genes (intragenic). Intergenic recombination is evident from inconsistencies between gene trees [34-36]. Intragenic recombination has been observed within the genes *wsp*, *ftsZ*, and *gltA* and within and between supergroups A and B [34,37-41]. Recently, a genomic comparison of A-group *Wolbachia* strains by Klasson et al. [42] showed highly recombining genomes, implying frequent horizontal gene transfer.

*Cardinium* has been less well investigated: although there is some evidence for inconsistent phylogenies between *Cardinium* and their hosts, which is compatible with horizontal transmission, only a few studies have been performed, mainly focusing on a single gene (16S rDNA) [2,15,18,21,23], and there is currently no evidence regarding recombination in *Cardinium*.

Phylogenetic and evolutionary studies on *Wolbachia* have mainly focused on samples representing a wide range of host species [26,34,37,38,43,44]. Based on two genes, Jiggins [38] showed that among strains from a wide range of host species, the rate of recombination is similar to that of a horizontally transmitted bacterium (*Cowdria ruminantium*). It remains however unclear to what extent these conclusions will be supported by the analyses of much more tightly defined samples such as those recovered from closely related host genera, or even from a single host species from a single geographical and temporal source. Most current studies which address this have used only one or two genes or a restricted number of species or populations [31,36,41,45]. A study by Baldo *et al. *[22] included a more detailed study of the extent of recombination and horizontal transfer in a single spider genus and revealed that horizontal transfer explains a large part of the *Wolbachia* distribution patterns within the genus. Exact rates of recombination among *Wolbachia* strains have however not been inferred so far, which makes it difficult to draw direct comparisons with rates found for other bacteria. Recombination rates can be obtained from multilocus sequence data. Strains that differ at only a single locus are grouped into clonal complexes. Subsequently, the allele sequences are examined to determine whether single allelic variants within a clonal complex result from point mutation or homologous recombination [46].

We present here a detailed study of the diversity of *Wolbachia* and *Cardinium* in the phytophagous spider mite family Tetranychidae, by analyzing strains recovered from seven *Bryobia* species, *Tetranychus urticae*, and *Petrobia harti*. We consider strain diversity between tetranychid host species, within single host species (investigating multiple populations; up to 20 populations for *B. kissophila*) and within single populations and individuals. Both *Wolbachia* and *Cardinium* have been reported from this family. *Wolbachia* has been detected in at least six asexual and one sexual *Bryobia* species and strains from both supergroup B and K have been found [12,47,48]. Supergroup K is a new supergroup that has only been detected in *Bryobia* so far [12]. We investigate intra- and intergenic recombination in *Wolbachia* (four genes) and *Cardinium* (two genes), and quantify the rate of recombination relative to mutation for *Wolbachia*, by analyzing the variation between pairs of very closely related strains. We compare this endosymbiont diversity to the degree of host congruence (co-speciation), host mitochondrial DNA diversity, and geographical distribution.

## Results

**Figure 1:**
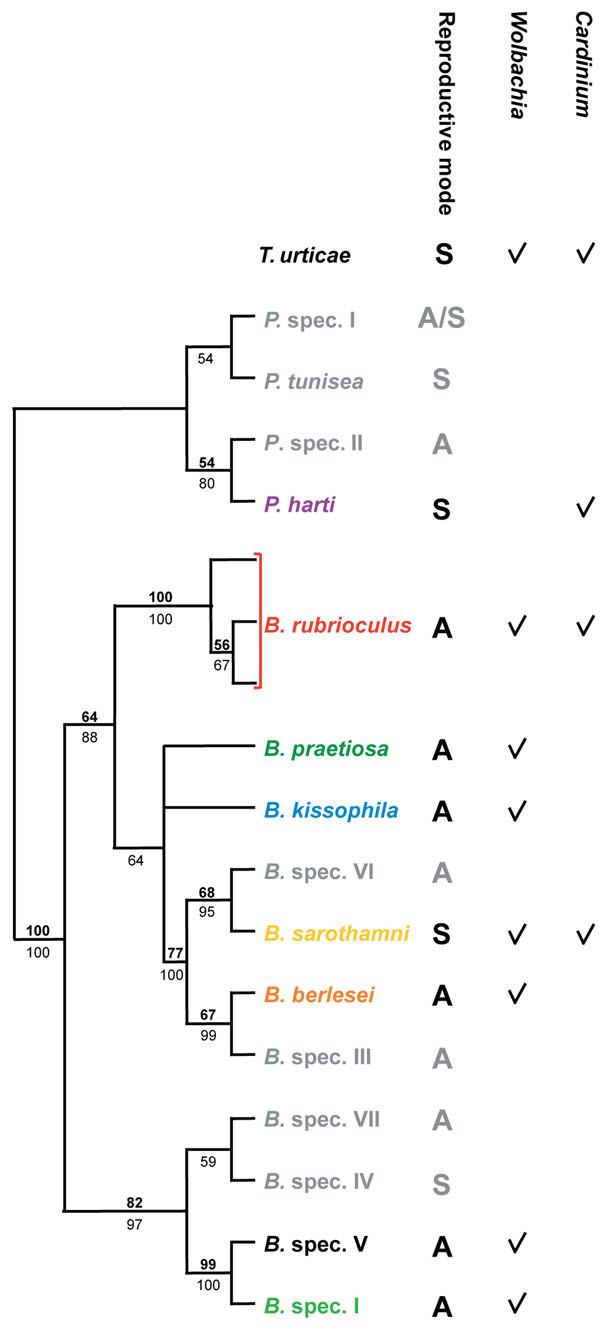
**Phylogenetic relationship between the tetranychid host species from which *Wolbachia* and *Cardinium* strains were obtained**. Maximum likelihood cladogram (28S rDNA) of the genus *Bryobia* and four outgroup species of the genus *Petrobia* is shown [49]. *Tetranychus urticae* was depicted separately as the exact position of *T. urticae* relative to the other host species was not studied so far. The genus *Tetranychus* belongs to another subfamily (Tetranychinae) than *Bryobia* and *Petrobia* (both Bryobiinae) of the family Tetranychidae. The mode of reproduction is given for each host species (A=asexual, S=sexual) in a separate column, and the subsequent columns indicate from which host species *Wolbachia* and or *Cardinium* strains were included in this study. Species names are colored as in Figure 2, 4, 5, and Additional file 3. Host species in grey were not included in this study. Numbers above branches (bold) indicate ML bootstrap values based on 1,000 replicates, numbers below branches (plain) depict Bayesian posterior probabilities (only values larger than 50 are indicted).

We included *Wolbachia* strains from seven *Bryobia* species (*B. berlesei*, *B. kissophila*, *B. praetiosa*, *B. rubrioculus*, *B. sarothamni*, *B.* spec. I, and *B.* spec. V) and *T. urticae*, and *Cardinium* strains from *B. rubrioculus*, *B. sarothamni*, *T. urticae*, and *P. harti* (Figure 1 and Additional file 1) [49]. We were unable to reliably determine the infection status of the other *Bryobia* host species (Figure 1) due to the lack of adequate material and/or inconsistent amplification of the bacterial genes, therefore these species were excluded from further analyses. The dataset includes strains from sexually (*B. sarothamni*, *T. urticae*, *P. harti*) and asexually (the remaining species) reproducing species.

**Figure 2:**
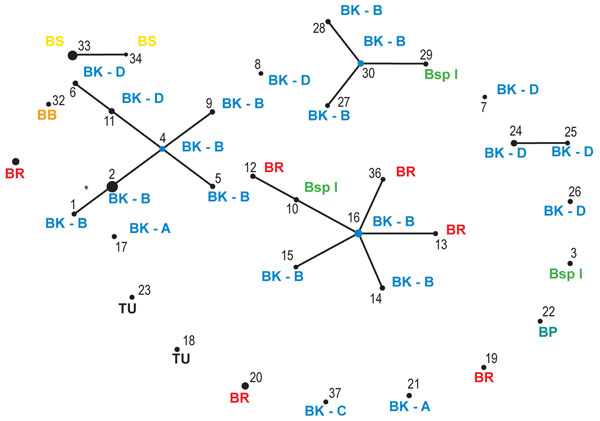
**Schematic overview of the clonal relatedness of the *Wolbachia* STs as predicted by eBURST.** Each ST is represented by a black dot, the size of which is proportional to the number of strains of that ST. STs that differ at a single locus are linked by lines. Only one variant is likely due to a mutational event (indicated by *), the other variants are most likely due to recombination events. STs that are not linked to other STs do not share at least four identical alleles with any other ST. Host species name in which each ST was detected is indicated: BB=*B. berlesei*; BK=*B. kissophila* (A-D indicate different COI clades, see text); BP=*B. praetiosa*; BR=*B. rubrioculus*; BS=*B. sarothamni*; BspI= *B.* spec. I; TU=*T. urticae*.

**Figure 3:**
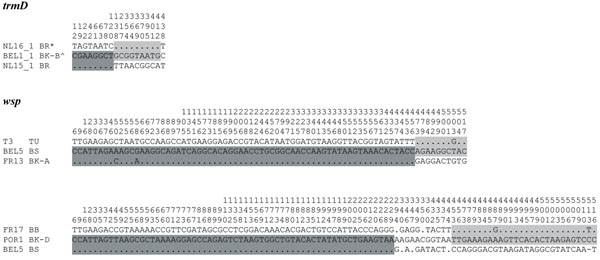
**Examples of recombination within *trmD* and *wsp.*** Only polymorphic sites are shown (position in alignment is given on top). Sequences are named by their sample code (Additional file 1) and abbreviated host species name (see legend Figure 2). Each sequence may have been found in different populations or host species, see phylogenies of *trmD* and *wsp* in Additional file 3. Different shadings indicate possible recombinant regions (see results). Differences and identities (dots) compared to the middle sequence are shown. * = also detected in BspI, BK-A, BK-C, and BP. ^ = also detected in BR.

**Figure 4:**
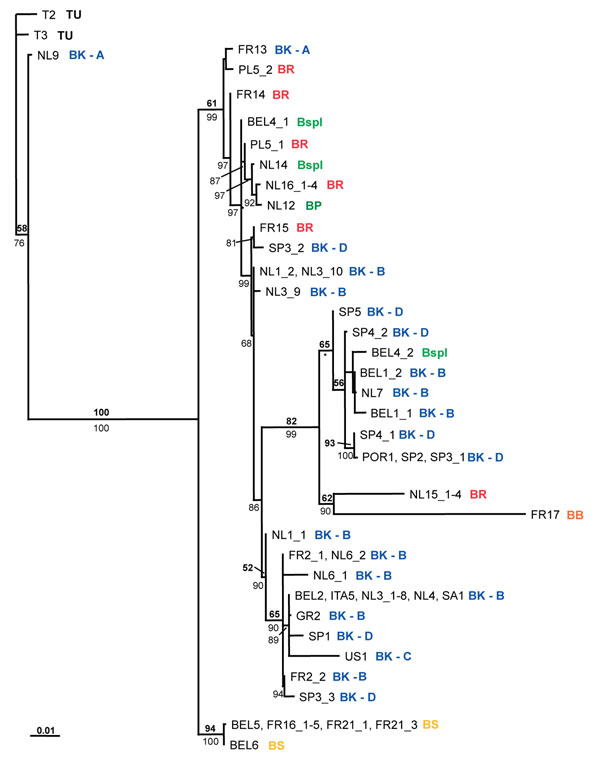
**Concatenated ML phylogeny for *Wolbachia*, based on four genes (*wsp*, *ftsZ*, *groEL*, and *trmD*)**. Thirty-six unique strains are shown. Sample code (Additional file 1) and host species name in which each strain was detected are indicated (for abbreviations see legend Figure 2). ML bootstrap values (top number, bold) and Bayesian posterior probabilities (bottom number, plain) are depicted (only values larger than 50 are indicated). * = the topology within this clade is slightly different for the MrBayes topology. The bar at the bottom indicates a branch length of 10% likelihood distance. Independent phylogenies for each gene are depicted in Additional file 3.

**Figure 5:**
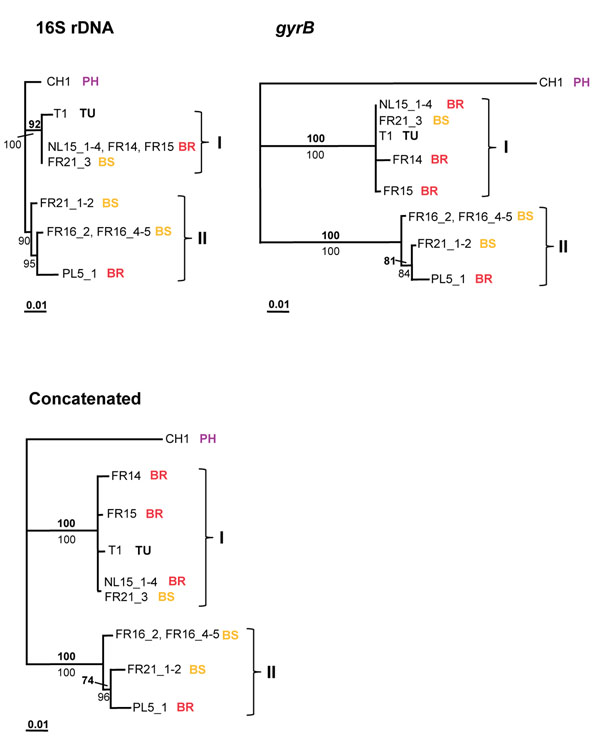
**16S rDNA, *gyrB*, and concatenated ML phylogenies for *Cardinium***. Sample code (Additional file 1) and host species name in which each strain was detected are indicated**:** BR=*B. rubrioculus*; BS=*B. sarothamni*; PH= *P. harti*. Two clades are named I and II. ML bootstrap values (top number, bold) and Bayesian posterior probabilities (bottom number, plain) are depicted (only values larger than 50 are indicated). The bar at the bottom indicates a branch length of 10% likelihood distance.

### Multiple infections

*Wolbachia* and *Cardinium* were found co-infecting *B. rubrioculus*, *B. sarothamni*, and *T. urticae*. In *B. rubrioculus* and *B. sarothamni*, *Wolbachia* and *Cardinium* strains were obtained from doubly infected individuals, whereas in *T. urticae* they were obtained from singly infected individuals (Additional file 1). Multiple *Wolbachia* strains infecting a single host individual were not detected, and neither were multiple *Cardinium* strains. Sequence chromatograms revealed no double peaks and cloning and sequencing of eleven PCR products did not reveal multiple infections.

### *Wolbachia* diversity

Sequences from the four *Wolbachia* genes (*wsp*, *ftsZ*, *groEL*, and *trmD*) were recovered for 65 *Wolbachia* infected individuals, except for *wsp* from *B.* spec. V (ITA11). The *Wolbachia* strain infecting *B.* spec. V belongs to the newly described supergroup K [12], which is highly divergent from supergroup B strains infecting other tetranychid mites. We excluded the supergroup K strain from phylogenetic and recombination analyses. No insertions or deletions were found within *ftsZ*, *groEL*, and *trmD*. Within *wsp* small indels (3-9bp) were found in a few strains but all sequences could be unambiguously aligned.

The sequenced *Wolbachia* strains reveal a high diversity. From the 64 *Wolbachia* strains (excluding the supergroup K *Wolbachia* strain in *B.* spec. V), 36 strains (sequence types; STs) were found unique (Additional file 2). Between 11 (*groEL*) and 18 (*trmD*) alleles were found per locus (Table 1). Nucleotide diversity was 5-11 times higher for *wsp* than for the other loci (Table 1). The dN/dS ratio was < 1 for all loci, indicating that the genes where not subjected to positive selection. The *wsp* gene also revealed a high rate of intragenic recombination (see below), with two sites identified within hyper variable region 1 (HVR1) under positive selection (HyPhy: codons 20 and 30; unpublished data). Despite this high nucleotide diversity and recombination rate for *wsp*, we found the most alleles for *trmD*.

**Table 1:** Diversity observed for four *Wolbachia* genes and two *Cardinium* genes.

	Locus	Size (bp)	Alleles	Variable sites		π	p-distance max. (%)	dN/dS ratio
							
				n	%			
***Wolbachia*****(n=64)**	*wsp*	525	13	155	29.52	0.1030	20.08	0.60
	*ftsZ*	507	14	20	3.94	0.0126	2.37	0.07
	*groEL*	491	11	18	3.67	0.0087	1.83	0.29
	*trmD*	453	18	34	7.51	0.0176	4.42	0.23

***Cardinium*****(n=15)**	CLO	407	6	15	3.69	0.0151	2.22	-
	*gyrB*	631	8	127	20.13	0.0839	14.9	0.06

π = nucleotide diversity

Forty-four out of the 64 strains were grouped into five clonal complexes (I-V; Figure 2 and Table 2). All other strains differed at more than one locus from the strains in these complexes. A total of 17 alleles deviated from the alleles from the founding genotypes within the clonal complexes (Table 2). A significant higher number of these variant alleles were found for *trmD* compared to the other loci (Table 2; Chi-square test; *p*=0.003), which is consistent with the observation that this locus contains the most alleles.

**Table 2:** Clonal complexes found for*Wolbachia*

Complex	I							II				III							IV		V	
					
ST^a^	4	9	5	2	1	11	6	30	29	28	27	16	15	14	13	36	10	12	24	25	33	34
*wsp*	1	-	-	-	-	-	5 ^18^	12	-	-	-	5	3 ^8^	-	-	-	-	4 ^16^	12	-	6	-
*ftsZ*	2	-	-	-	**1 ^1^**	-	-	10	-	-	-	3	-	-	-	-	-	-	10	-	14	-
*groEl*	8	-	-	4 ^4^	4 ^4^	-	-	8	-	-	-	8	-	4 ^4^	-	-	-	-	12	*11 ^2^*	3	-
*trmD*	1	3 ^1*^	10 ^15^	-	-	6 ^7^	6 ^7^	1	14 ^9^	6 ^7^	2 ^1*^	1	-	-	5 ^2*^	17 ^9^	15 ^8^	15 ^8^	8	-	9	11 ^1*^
Species^b^	BK-B	BK-B	BK-B	BK-B	BK-B	BK-D	BK-D	BK-B	BspI	BK-D	BK-B	BK-D	BK-D	BK-D	BR	BR	BspI	BR	BK-D	BK-D	BS	BS
Freq.^c^	2	1	1	12	1	1	1	1	1	1	1	2	1	1	1	1	1	1	3	1	8	1

^a^ Allelic variants within each clonal complex are depicted, listed per sequence type (ST). Likely recombinational changes are depicted in plain text, and putative mutations are shown in bold. Disputable cases are highlighted in italics (possible recombinational changes). For each allelic variant the allele number is given, with in superscript the number of polymorphic sites between the allelic variant and the typical allele of the clonal group. * indicates non-unique mutations (in cases where one or two mutations were found).

^b^ Host species name in which each ST was detected is indicated (for abbreviations see legend Figure 2).

^c^ The frequency of each sequence type is listed.

#### Recombination between *Wolbachia* strains

We investigated intergenic recombination by analysis of allelic variation within the clonal complexes. This approach reveals whether variant alleles arose by point mutation or by recombination. Of the 17 variant alleles, four differed from the typical allele in the clonal complex by a single nucleotide change (Table 2). Three of these single nucleotide changes, however, were non-unique. Two other alleles differed by two nucleotide changes, and could be either derived by point mutations or recombination (the chance of two independent mutations both occurring in one out of four genes is 0.25). One of these variant alleles was found elsewhere in the dataset, implicating recombination. All other allelic variants differed from the founder alleles at four or more sites and were considered as putative recombinational imports. Ignoring alleles with one non-unique and two nucleotide changes, the estimated ratio of recombinational events to mutational events per gene fragment is 11:1. If we include non-unique changes as recombinational imports, and unique changes as point mutations, the ratio is 15:2. We therefore conclude that new alleles were 7.5 to 11 times more likely to be generated by recombination than by point mutation. This is a conservative estimate because single nucleotide changes were attributed to point mutation and not to recombination, although recombination between similar alleles could result in a single nucleotide change. Further, a high rate of recombination is consistent with the observed incongruence between the four gene tree topologies (Additional file 3).

Intragenic recombination is another process that may contribute to the origin of new *Wolbachia* genotypes. We detected intragenic recombination within the *trmD* and *wsp* genes (Figure 3). The alignment of *wsp* genes shows that the polymorphic sites are not randomly distributed, but clearly shows a mosaic pattern consistent with recombination. Intragenic recombination is not restricted to *Wolbachia* strains from the same host species, but also involves strains infecting different host species. For example, the *wsp* sequence obtained from *Wolbachia* in *B. sarothamni* (all populations) is a recombinant between the *wsp* sequences obtained from *Wolbachia* in *B. kissophila* (FR13) and *T. urticae* (T3) (Figure 3).

#### Cospeciation of *Wolbachia* and host species

Examination of the concatenated *Wolbachia* phylogeny reveals that there is generally a lack of cospeciation between host and parasite (Figure 4). *Wolbachia* strains obtained from a single host species do not clearly cluster. For example, strains from *B. rubrioculus* are found at different places in the phylogeny. The same is true for strains from *B.* spec. I. On the other hand, the *Wolbachia* phylogeny is not completely random with respect to host species. Some *Wolbachia* strains from *B. kissophila* cluster together, whilst others cluster with strains from *B.* spec I (BEL4_2) or *B. rubrioculus* (FR15). Two *B. kissophila*-derived strains (NL9 and FR13) are very divergent from all other *B. kissophila* strains. In the exceptional case of *B. sarothamni*, the same *Wolbachia* genotype was found in all five populations (from Belgium and France; except for a minor difference in *trmD* for BEL6; Figure 2, 4, and Table 2). This strain was not found in any of the other species, although it closely resembles the *Wolbachia* strain infecting *B. berlesei* at three of the four genes (*wsp* is highly divergent between the two strains). *Bryobia sarothamni* and *B. berlesei* share the same host plant species, *Cytisus scoparius*, which potentially facilitates horizontal transmission of both *Wolbachia* strains and genes.

Finally, a single *B. praetiosa* individual was investigated. Although this species was found to harbor a unique *Wolbachia* strain, this strain shares each of its alleles with strains in (multiple) other host species. Although allelic identity by descent cannot be ruled out without more detailed analysis, this observation is also consistent with frequent inter-allele recombination. Within the other species, divergent *Wolbachia* strains were found between populations and also within populations (Figure 4). In five *B. rubrioculus* mite populations, six divergent *Wolbachia* strains were found: population PL5 contains two divergent *Wolbachia* strains. For *B*. spec. I three *Wolbachia* strains were detected in two populations: two individuals from BEL4 harbor highly divergent *Wolbachia* strains (mainly due to differences at *wsp* and *ftsZ*).

#### Correlation between *Wolbachia* and host mitochondrial diversity or geographical location

It has been suggested that infection by *Wolbachia* affects host mitochondrial diversity and that mitochondrial haplotypes and *Wolbachia* haplotypes may be linked [50-53]. As this has serious implications for population studies based on mtDNA [54], we were motivated to examine this possibility for *B. kissophila*. High levels of diversity at the mitochondrial COI locus were observed within *B. kissophila*, which resolved into four clades (A-D) [49]. However, there was little evidence for correlation between the COI haplotypes and the *Wolbachia* strains (Figure 2 and 4). A total of 20 populations were investigated for *B. kissophila*, and a highly divergent set of *Wolbachia* strains was found within this species. Twenty-one *Wolbachia* strains were found, four of which were shared between populations. Within several populations (BEL1, FR2, NL1, NL3, NL6, SP3, and SP4) more than one *Wolbachia* strain was detected.

*Bryobia kissophila* COI clade A was highly divergent from all other COI clades, and contains *Wolbachia* strains that are divergent from the ones found in the other clades. However, the two investigated populations belonging to clade A (NL9 and FR13) harbor divergent *Wolbachia* strains. Also, some alleles of these strains are shared with other *B. kissophila* clades (for *groEL* and *trmD*) or with other *Bryobia* species (for all four genes) (Additional file 3). *Wolbachia* strains from clade B, C, and D show a mixture of different *Wolbachia* strains. There is no correlation with COI haplotype, although there are no strains shared among populations belonging to different COI clades.

There is a similar lack of congruence between *Wolbachia* strain diversity and geographic location of the host populations. Very distant populations may harbor identical *Wolbachia* strains (e.g., BEL2 and SA1; *B. kissophila*), while nearby populations harbor very divergent *Wolbachia* strains (e.g., NL15 and NL16; *B. rubrioculus*). Also within populations divergent strains are found.

### *Cardinium* diversity

*Cardinium* diversity was investigated by sequencing part of the 16S rDNA and *gyrB* gene. Sequences were successfully recovered from all *Cardinium* infected individuals and all sequences could be unambiguously aligned. No insertions or deletions were found within *gyrB*. Within 16S rDNA, one insertion and one deletion (both 1bp) were found. For 16S rDNA six alleles were found, with 3.7% variable sites, a maximum p-distance of 2.2%, and a nucleotide diversity of 0.015 (Table 1). Diversity for *gyrB* was much higher, with eight alleles, 20.1% variable sites, a maximum p-distance of 14.9%, and a nucleotide diversity of 0.084.

In total, eight strains were detected within eight populations, belonging to four mite species, and phylogenetic analysis resolved these eight stains into two major clades (Figure 5). The *Cardinium* strain found in *P. harti* (CH1) is divergent from two other clades (named I and II), which were detected in *B. sarothamni* and *B. rubrioculus* (both clade I and II), and in *T. urticae* (clade I). These two clades are highly supported. Clade I and II differed at 1.7% of nucleotide sites for 16S rDNA and at 10.6% for *gyrB*, while differences within clades are small (<1.2% for both genes). Generally, there is congruence between the phylogenies obtained for 16S rDNA and *gyrB* which suggests less recombination than in *Wolbachia*, although the evidence is equivocal*.* However, there is no obvious association between *Cardinium* genotype and host species. Clade I contains strains found in three *B. rubrioculus* populations and in one *T. urticae* and one *B. sarothamni* population, while clade II contains highly related strains found in two *B. sarothamni* populations and one *B. rubrioculus* population. One strain was found infecting two host species: *B. rubrioculus* (NL15_1-4) and *B. sarothamni* (FR21_3). Other strains belonging to *B. sarothamni* population FR21 group within clade II (FR21_1-2). These patterns imply horizontal transfer of strains (or genes) between and within host species.

## Discussion

This detailed study of reproductive parasites in nine tetranychid mite species reveals a high genetic diversity. *Wolbachia* strains belonging to two highly divergent supergroups (B and K) were detected (see also [12]). The diversity within supergroup B was high, with 36 unique strains found in 64 investigated individuals. The level of recombination detected is extremely high, supporting the mosaic genome structure of *Wolbachia *[42]. *Cardinium* was less frequently found in the mites than *Wolbachia*, but also showed a high level of diversity, with eight unique strains detected in 15 individuals on the basis of only two genes.

### *Wolbachia* diversity

We investigated *Wolbachia* diversity at a fine scale with respect to host diversity, by comparing strains from nine closely related host species, all belonging to the family Tetranychidae, and mainly from the genus *Bryobia*. Our study shows that even within a single host genus there exists a high level of *Wolbachia* diversity. *Wolbachia* strains belonging to two highly divergent supergroups (B and K) were detected. Even within *Wolbachia* supergroup B, 36 unique STs were obtained from 64 infected hosts. Although there was little correlation between host species and *Wolbachia* strains, strains were not distributed randomly among different species (Figure 2 and 4), so that a certain level of specificity was observed. Strains within clonal complex I were restricted to *B. kissophila* and within clonal complex V to *B. sarothamni*. Other complexes however contain strains from different host species. It is striking that many alleles are shared among the different STs, even from different host species, indicating that recombination contributes substantially to the genetic diversity of *Wolbachia*. Recombination is further evidenced by the many phylogenetic conflicts observed among the individual gene trees and a high recombination rate compared to mutation rate. Analysis of the variant alleles in the clonal complexes reveals that the rate of recombination compared to point mutation in the diversification of lineages ranges between 7.5:1 and 11:1.

The observed recombination rate and diversity is much higher than what would be expected for clonal organisms. Recombination is rare in other clonally inherited, obligate intracellular bacteria [55,56]. The high recombination rate we found is comparable to rates of horizontally transmitted human pathogens. For example, for *Streptococcus pneumoniae* a recombination to mutation ratio of 10:1 was found, for *Neisseria meningitidis* a ratio of 5:1 [57]. Horizontal transmission of *Wolbachia* has been observed, but examples are rare [30-32]. Although many studies based on molecular data have suggested extensive horizontal gene transfer of *Wolbachia *[22,25,35,36,42,43], it is unclear if bacteria are transmitted horizontally, or if the transfer concerns single genes, possibly via bacteriophages [58]. The high rate of recombination found in this study, the observation that individual alleles are shared among *Wolbachia* strains from different host species but complete STs are not, and the fact that *Wolbachia* is mainly clonally inherited, suggest that individual genes rather than complete bacteria are exchanged. Alternatively, transfer of bacteria leading to mixed infections and subsequent recombination may explain these observations. Although our cloning data suggest that mixed infections are rare, this possibility cannot be excluded (see also [59]). The observation that the trees are not completely random with respect to host species suggests that vertical transmission does occur [26,43].

Homologous recombination in bacteria can occur by transformation, conjugation, or transduction. Conjugation and transformation require physical contact, or close proximity, of donor DNA and recipient bacteria. Ecological circumstances may create opportunities for recombination, e.g., *Wolbachia* strains from *B. sarothamni* and *B. berlesei* share three of the four alleles, and their mite hosts feed on the same host plant species (we found both species co-occurring on the same individual plant). Other ecological interactions have been suggested as means for bacteria or gene exchange, e.g., host-parasite interactions or double *Wolbachia* infections [28,36,45]. However, in many other cases, opportunities for recombination are less obvious. Transduction involving vectors (e.g., plasmids, phages, or viruses) is a more likely manner of gene exchange. Good vector candidates are bacteriophages, as these have been isolated from *Wolbachia* infected populations [60-62] and seem to be common in *Wolbachia* genomes [42,63]. Phylogenetic analyses suggest that the bacteriophage WO is horizontally transferred between different *Wolbachia* strains, and is able to infect new *Wolbachia* hosts [60,61,64]. Other, free-living, bacteria might even be involved in phage-transfer. We also noted the presence of a bacteriophage in an individual of *B.* spec. I. The bacteriophage sequence, detected coincidentally with *groEL* primers, appeared similar to the sequence of the *Wolbachia* bacteriophage WOcauB1 from *Cadra cautella* (GenBank: AB161975; 12% p-distance) [65], and to part of the sequenced genome (located within the gene *dnaA*) of *Wolbachia* from *Drosophila melanogaster* (GenBank: AE017196; 11% p-distance).

With strict vertical transmission, strong linkage disequilibrium between host mtDNA and *Wolbachia* would be expected. However, recombination may uncouple such associations, and could be a reason for the observed lack of congruence between host mtDNA and *Wolbachia* STs. There are some signs of congruence, with related host strains (with identical COI sequences) sharing identical or closely related *Wolbachia* strains, but due to the high rate of recombination such associations are broken up rather quickly.

### *Cardinium* diversity

For *Cardinium*, the two investigated genes showed highly similar phylogenies, giving no clear evidence for intergenic recombination. Also, no signs of intragenic recombination were found. There was however no congruence between *Cardinium* strains and associated host species: similar strains were found in *B. rubrioculus*, *B. sarothamni*, and *T. urticae*. Only the strain infecting *P. harti* was clearly distinct from all other strains. The sharing of strains among different host species, and the occurrence of divergent strains in one host population (FR21), suggest that horizontal transmission is also prevalent for *Cardinium*. Horizontal transmission seemed also to explain diversity patterns found for *Cardinium* infecting *Cybaeus* spiders [17]. Patterns of recombination and horizontal transfer should however be further studied including more genes. An MLST set for *Cardinium* is desirable, for reliable strain typing and for investigating patterns of recombination, horizontal transmission, or host manipulation. This requires the use of several independent markers, sufficiently distant from each other within the genome.

### Phenotypic effects of *Wolbachia* and *Cardinium* in spider mites

We analyzed *Wolbachia* and *Cardinium* strains from both asexual and sexual host species. Weeks and Breeuwer [48] showed that *Wolbachia* is involved in causing asexuality in at least two species: *B. praetiosa* and an unidentified species. *Wolbachia* is possibly causing asexuality in the other infected asexual *Bryobia* species as well. The general observation is that all individuals within the asexual *Bryobia* species are infected with *Wolbachia*. No males have ever been observed, neither in cultures nor in the field, and additional lab experiments including at least 20 individuals per species (except for *B. berlesei*) show a fixed infection with *Wolbachia* (unpublished data). Moreover, Weeks and Breeuwer [48] analyzed 240 *B. kissophila*, 144 *B. praetiosa*, and 24 *B. rubrioculus* individuals and found all individuals infected with *Wolbachia*. We detected *Cardinium* in one asexual species, *B. rubrioculus*. This species is doubly infected with both *Wolbachia* and *Cardinium*, although *Cardinium* was not found in all individuals. It is unclear if *Cardinium* is having an effect on the host species, but it is unlikely that it induces the asexuality as not all individuals are infected. We detected both *Wolbachia* and *Cardinium* in the sexually reproducing species *B. sarothamni* and *T. urticae*. Both species appear polymorphic for infection with both bacteria. *Cardinium* induces strong CI in *B. sarothamni*, while no effect for *Wolbachia* has been found so far [47]. Previously, *Wolbachia* was found inducing CI in *T. urticae *[66-69], but no effect of *Cardinium* on *T. urticae* was found so far [68]. We detected only *Cardinium* in *P. harti*, but Weeks *et al. *[2] also report *Wolbachia* from *P. harti*. The effects of both *Wolbachia* and *Cardinium* in *P. harti*, and *T. urticae* require further investigation.

## Conclusions

We found a relatively high rate of recombination for *Wolbachia* strains obtained from host species of the family Tetranychidae. Considering the fact that *Wolbachia* is widely distributed among arthropods, we investigated strains from a restrictive host range. It remains to be investigated if our findings present a general pattern and if similar recombination rates will be found among strains from other restricted host ranges. Our study of diversity within *Cardinium* revealed incongruencies among host and bacterial phylogenies, confirming earlier findings. Analysis of additional genes is needed to investigate recombination rates within this reproductive parasite.

## Methods

### DNA isolation, amplification, and sequencing

We analyzed *Wolbachia* and *Cardinium* strains from seven *Bryobia* species (34 populations), *T. urticae* (three populations), and *P. harti* (one population) (Figure 1 and Additional file 1). Samples were collected between May 2004 and November 2006 from eight European countries, and from South Africa, the United States, and China. For each host population, information on mitochondrial (part of the COI gene) and nuclear (part of the 28S rDNA gene) diversity was obtained as described in Ros *et al. *[49] and used for species identification. Mites were either set up as cultures in the lab or stored in 96% ethanol. DNA was extracted from single mites using the CTAB extraction method as previously described [54] or using the NucleoSpin Kit (Macherey-Nagel, Düren, Germany) following manufacturers’ instructions.

For *Wolbachia*, four genes were amplified and sequenced: *wsp*, *flsZ*, *groEL*, and *trmD. Wsp* was amplified and sequenced using the primers *wsp*-81F and *wsp-*691R [70]. *FtsZ* and *groEl* were amplified and sequenced as described in Ros *et al. *[49]. *TrmD* was amplified and sequenced using the primers *trmD-*F 5’-GAACTATTCTCTTTGCCGGAAAAGC-3’ and *trmD-*R 5’-CACTGCTCAGGTCTAGTATATTGAGG-3’.These primers were designed from available *Wolbachia* and *Rickettsia* genome sequences [71-73] and were shown to reliably amplify products from strains representative of supergroups A and B (data not shown; samples kindly donated by Dr. Robert Butcher).

For *Cardinium*, two genes were amplified: 16S rDNA and *gyrB.* 16S rDNA was amplified and sequenced directly using the primers CLOf and CLOr1 [2]. *GyrB* was amplified using primers from Groot and Breeuwer [74], cloned, and subsequently sequenced. Amplified fragments were separated from non-specific products by running the PCR products on a 1% agarose in 1x TAE gel and excising the fragments from the gel. Fragments were purified using the method of Boom *et al. *[75]. Products were first cloned and subsequently sequenced following the cloning protocol described below, with 1-2 clones sequenced per sample using M13 forward and reverse primers.

PCR amplifications were performed in 25 μl reactions containing 1X Super Taq buffer (HT BioTechnology, Cambridge, UK), 0.5 mg/ml bovine serum albumin (BSA), 1.25 mM MgCl_2_, 0.2 mM dNTP’s, 160 nM of each primer, 1 u of Super Taq (HT BioTechnology), and 2.5 μl of DNA extract. For *ftsZ*, *groEL*, and *trmD*, no MgCl_2_ was added and for 16S rDNA no MgCl_2_ and BSA was added. PCR cycling profile for *wsp* and *ftsZ* was 35 cycles of 30 sec. at 95 °C, 30 sec. at 51 °C, and 1 min. at 72 °C, for *groEL* and *trmD* 35 cycles of 1 min. at 95 °C, 1 min. at 49 °C, and 1.5 min. at 72 °C, for *Cardinium* 16S rDNA 35 cycles of 40 sec. at 95 °C, 40 sec. at 57 °C, and 45 sec. at 72 °C, and for *gyrB* 35 cycles of 1 min. at 95 °C, 1 min. at 50 °C, and 1 min. at 72 °C. Products (2 μl) were visualized on a 1% agarose gel stained with ethidium bromide in 0.5X TBE buffer (45mM Tris base, 45mM boric acid, and 1 mM EDTA, pH 8.0). PCR products were purified using a DNA extraction kit (Fermentas, St. Leon-Rot, Germany). The purified products were directly sequenced using the ABI PRISM BigDye Terminator Sequence Kit (Applied Biosystems, Nieuwerkerk a/d IJssel, The Netherlands). Both strands of the products were sequenced using the same primers as used in the PCR amplification. Sequences were run on an ABI 3700 automated DNA sequencer.

All new unique sequence data have been submitted to the GenBank under accession numbers: JN572802-JN572888 (see Additional file 4).

### Testing for multiple infections

We tested for multiple infections by checking all sequences for the presence of double peaks in the chromatograms and for differences between forward and reverse sequences. Additionally, 11 PCR products were cloned and subsequently sequenced (two for *wsp*, *groEL*, *trmD*, and *gyrB*, and three for *ftsZ*) (Additional file 1). This approach would reveal multiple infections by *Wolbachia* or *Cardinium*. PCR products selected for cloning were cleaned using the method of Boom *et al. *[75]. The cleaned products were ligated into vectors and transformed into bacteria using the pGEM-T Easy Vector System and JM109 competent cells (Promega, Madison WI, US). Plasmids were recovered for 3-11 colonies per sample, using mini-preparation procedures [76]. Plasmids were sequenced using the M13 forward and reverse primers.

### Data assembling and phylogenetic analyses

Sequences were aligned using ClustalX version 1.8.0 with default settings [77] and modified in BioEdit version 7.0.7 [78]. We excluded one *Wolbachia* strain (ITA11) from subsequent analyses, as this strain represents a separate supergroup and is highly divergent from all other strains (see results). We analyzed alignments of 525bp for *wsp*, 557bp for *ftsZ*, 491bp for *groEL*, 453bp for *trmD*, 407bp for *Cardinium* 16S rDNA, and 631bp for *gyrB*. Nucleotide diversity was calculated in MEGAv4.0 [79]. The program SNAP (http://www.hiv.lanl.gov) [80] was used to calculate the rate of nonsynonymous to synonymous substitutions (dN/dS). To determine the overall selection pressures faced by each gene, the SLAC method within the HyPhy package was used [81]. Phylogenetic analyses were performed using Neighbor-Joining (NJ), Maximum Likelihood (ML), and Bayesian methods, for each gene separately and for a concatenated dataset of four genes for *Wolbachia* and two genes for *Cardinium*. PAUP* version 4.0b10 [82] was used to select the optimal evolution model by critically evaluating the selected parameters [83] using the Akaike Information Criterion (AIC) [84]. For the protein coding genes, we tested if the likelihood of models could be further improved by incorporating specific rates for each codon position [85]. This approach suggested the following models: *wsp* (submodel of GTR + G with rate class 'a b c c a c'),*ftsZ* (K3P+I), *groEL* (submodel of GTR with rate class 'a a b b a b'), *trmD* (HKY with site-specific rates for each codon position), 16S rDNA (submodel of GTR with rate class 'a a b c a c'), *gyrB* (submodel of GTR with rate class 'a b c d b a' and site-specific rates), the concatenated *Wolbachia* dataset (submodel of GTR + I + G with rate class ‘a b c c b d’), and the concatenated *Cardinium* dataset (submodel of GTR + G with rate class ‘a b c a b c’). Under the selected models, parameters and tree topology were optimized using the successive approximations approach [86]. NJ analyses (p-distances) and ML analyses (heuristic search, random addition of sequences with 10 replicates, TBR branch swapping) were performed in PAUP. Robustness of nodes was assessed with 100 NJ- resp. ML-bootstrap replicates. However, as PAUP does not allow for site-specific rates in bootstrap analysis, ML bootstrapping for *trmD* and *gyrB* was performed with gamma distributed rates, with 100 bootstrap replicates. Bootstrap values were then plotted on the phylogeny obtained with the original model with site-specific rates. Bayesian analyses were performed as implemented in MrBayes 3.1.2 [87]. Models used were GTR + G (*wsp*), GTR + I (*ftsZ*), GTR (*groEL*, 16S rDNA), and GTR with separate rates for each codon position (*trmD*, *gyrB*)*.* For the concatenated dataset, the same models were used for each gene partition. Analyses were initiated from random starting trees. Two separate Markov Chain Monte Carlo (MCMC) runs, each composed of four chains (one cold and three heated), were run for 6,000,000 generations (7,000,000 generations for the concatenated *Wolbachia* set). The cold chain was sampled every 100 generations, the first 15,000 generations were discarded afterwards (burn-in of 25%). Posterior probabilities were computed from the remaining trees. We checked whether the MCMC analyses ran long enough using the program AWTY [88]. Stationarity was assumed when there was convergence between the two MCMC runs and when the cumulative posterior probabilities of splits stabilized; in all analyses 6,000,000 generations proved sufficient. The concatenated *Wolbachia* dataset however, showed no convergence or stabilization of probabilities (not even after 15,000,000 generations). This is most likely due to the extensive recombination present within this dataset.

### Analysis of recombination

Evidence for recombination within *Wolbachia* and *Cardinium* was obtained by comparing topologies of different genes. For *Wolbachia*, we also quantified the relative impact of recombination compared to point mutation over short-term clonal diversification. Following standard MLST protocol [89], we assigned allele identifiers for each unique sequence at a particular locus, and an “ST” (sequence type) for each unique allelic profile. We used eBURST version 3 [90] (Figure 2) to identify closely related pairs or clusters (clonal complexes). All members assigned to a clonal complex share identical alleles at three of the four loci with at least one other ST member of the complex. By comparing, for each ST within a clonal complex, the sequence of the deviating allele with the allele of the founding genotype, it is possible to estimate how many STs have arisen by *de novo* point mutation (i.e. a novel change at a single base) or homologous recombination (a single non-unique change or multiple nucleotide changes) [46].

Additionally, single gene alignments for *Wolbachia* and *Cardinium* were checked for signs of intragenic recombination using the software package RDP3 [91] and by visual inspection. Programs used in the RDP3 software package were RDP, Geneconv, Bootscan, MaxChi, Chimaera, and Sister Scanning. Analyses were run with default settings, except for window- and stepsizes: these were varied during independent analyses. Analyses were performed for total datasets and reduced datasets (removal of highly similar strains). This analysis was performed for each of the four *Wolbachia* genes and for the two *Cardinium* genes.

## Authors’ contributions

VIDR collected samples, carried out the molecular studies and analyzed data. VMF carried out the molecular studies and analyzed data. VIDR, VMF, JAJB and EJF conceived the study and VIDR, JAJB and EJF drafted the manuscript. All authors read and approved the final manuscript.

## Competing interests

The authors declare that they have no competing interests.

## Supplementary Material

Additional file 1 List of tetranychid samples in which *Wolbachia* and/or *Cardinium* strains were detected. Click here for file

Additional file 2 Allelic profiles for each of the 37 unique *Wolbachia* STs. Click here for file

Additional file 3 *Wolbachia* gene phylogenies (*wsp*, *ftsZ*, *groEL*, and *trmD*). Click here for file

Additional file 4 GenBank accession numbers. Click here for file
