# Supplementary material for: Diversity and recombination in Wolbachia and Cardinium from Bryobia spider mites
Source: BMC Microbiol. 2012 Jan 18;12(Suppl 1):S13. doi: 10.1186/1471-2180-12-S1-S13 (PMC3287510; doi:10.1186/1471-2180-12-S1-S13)
Supplement: Additional file 1 — List of tetranychid samples in which Wolbachia and/or Cardinium strains were detected. [file 1471-2180-12-S1-S13-S1.pdf]

## Additional file 1 - List of tetranychid samples in which *Wolbachia* and/or *Cardinium* were detected

| Code | Species | Country | Locality | Host plant | | Collection date | n | Individual code | W | C | cloned | |
|---|---|---|---|---|---|---|---|---|---|---|---|---|
| | | | | Common name | Scientific name | | | | | | sample | gene |
| BEL1 | *B. kissophila* | Belgium | Lompret | Ivy | *Hedera helix* | May 2004 | 2 | | + | - | | |
| BEL2 | *B. kissophila* | Belgium | Olloy-sur-Viroin | Ivy | *Hedera helix* | May 2004 | 1 | | + | - | | |
| FR2 | *B. kissophila* | France | Angers | Ivy | *Hedera helix* | May 2004 | 2 | | + | - | | |
| FR13 | *B. kissophila* | France | Vireux | Ivy | *Hedera helix* | May 2004 | 1 | | + | - | | |
| GR2 | *B. kissophila* | Greece | Thermi | Ivy | *Hedera helix* | May 2005 | 1 | | + | - | | |
| ITA5 | *B. kissophila* | Italy | Rome | Ivy | *Hedera helix* | May 2005 | 1 | | + | - | | |
| NL1 | *B. kissophila* | Netherlands | Hardegarijp | Ivy | *Hedera helix* | April 2004 | 2 | | + | - | | |
| NL3 | *B. kissophila* | Netherlands | Amsterdam | Ivy | *Hedera helix* | April 2004 | 2 | NL3_9-10 | + | - | | |
| | | | | | | Nov 2006 | 8 | NL3_1-8 | + | - | NL3_1 | *trmD* |
| NL4 | *B. kissophila* | Netherlands | Den Burg (Texel) | Ivy | *Hedera helix* | June 2006 | 1 | | + | - | | |
| NL6 | *B. kissophila* | Netherlands | Valkenburg | Ivy | *Hedera helix* | May 2004 | 2 | | + | - | | |
| NL7 | *B. kissophila* | Netherlands | Varsseveld | Ivy | *Hedera helix* | May 2004 | 1 | | + | - | | |
| NL9 | *B. kissophila* | Netherlands | Nes (Ameland) | Ivy | *Hedera helix* | June 2006 | 1 | | + | - | | |
| POR1 | *B. kissophila* | Portugal | Portimao | Ivy | *Hedera helix* | Feb 2005 | 1 | | + | - | | |
| SA1 | *B. kissophila* | South Africa | Johannesburg | Ivy | *Hedera helix* | Aug 2005 | 1 | | + | - | | |
| SP1 | *B. kissophila* | Spain | Begues | Ivy | *Hedera helix* | April 2004 | 1 | | + | - | | |
| SP2 | *B. kissophila* | Spain | Vill. d'Escornalbou | Ivy | *Hedera helix* | April 2004 | 1 | | + | - | | |
| SP3 | *B. kissophila* | Spain | Gatova | Ivy | *Hedera helix* | April 2004 | 3 | | + | - | | |
| SP4 | *B. kissophila* | Spain | Alcossebres | Ivy | *Hedera helix* | April 2004 | 2 | | + | - | | |
| SP5 | *B. kissophila* | Spain | Alcossebres | Ivy | *Hedera helix* | April 2004 | 1 | | + | - | | |
| US1 | *B. kissophila* | United Sates | Riverside | Ivy | *Hedera helix* | May 2005 | 1 | | + | - | US1 | *groEL, ftsZ* |
| NL12 | *B. praetiosa* | Netherlands | Amsterdam | Grass and herbs | - | May 2004 | 1 | | + | - | NL12 | *wsp* |
| FR14 | *B. rubrioculus* | France | Aubrives | Apple | *Malus* spec. | May 2004 | 1 | | + | + | FR14 | *wsp* |
| FR15 | *B. rubrioculus* | France | Peumerit | Apple | *Malus* spec. | Aug 2006 | 1 | | + | + | | |
| NL15 | *B. rubrioculus* | Netherlands | Amsterdam | Apple | *Malus* spec. | May 2006 | 4 | | + | + | | |
| NL16 | *B. rubrioculus* | Netherlands | Amsterdam | Plum | *Prunus* spec. | May 2006 | 4 | | + | - | | |
| PL5 | *B. rubrioculus* | Poland | Krakow | Apple | *Malus* spec. | May 2006 | 2 | | | | | |
| | | | | | | | | PL5_1 | + | + | PL5_1 | *trmD, gyrB* |
| | | | | | | | | PL5_2 | + | - | | |

| Code | Species | Country | Locality | Host plant | | Collection date | N | | W | C | cloned | |
|---|---|---|---|---|---|---|---|---|---|---|---|---|
| | | | | Common name | Scientific name | | | | | | sample | gene |
| BEL5 | *B. sarothamni* | Belgium | Vierves sur Viroin | Common Broom | *Cytisus scoparius* | July 2006 | 1 | | + | - | | |
| BEL6 | *B. sarothamni* | Belgium | Vierves sur Viroin | Common Broom | *Cytisus scoparius* | May 2004 | 1 | | + | - | | |
| FR16 | *B. sarothamni* | France | Vireux | Common Broom | *Cytisus scoparius* | July 2006 | 5 | | | | | |
| | | | | | | | | FR16_1 | + | - | | |
| | | | | | | | | FR16_2 | + | + | | *gyrB* |
| | | | | | | | | FR16_3 | + | - | | |
| | | | | | | | | FR16_4 | + | + | | |
| | | | | | | | | FR16_5 | + | + | | |
| FR21 | *B. sarothamni* | France | Piriac sur mer | Common Broom | *Cytisus scoparius* | Aug 2006 | 3 | | | | | |
| | | | | | | | | FR21_1 | + | + | | |
| | | | | | | | | FR21_2 | - | + | | |
| | | | | | | | | FR21_3 | + | + | | |
| FR17 | *B. berlesei* | France | Vireux | Common Broom | *Cytisus scoparius* | May 2004 | 1 | | + | - | FR17 | *ftsZ* |
| BEL4 | *B.* spec. I | Belgium | Olloy-sur-Viroin | Vetches | *Vicia* spec. | May 2004 | 2 | | + | - | BEL4_1 | *groEL* |
| NL14 | *B.* spec. I | Netherlands | Nieuweschans | Vetches | *Vicia* spec. | May 2006 | 1 | | + | - | | |
| ITA11 | *B.* spec. V | Italy | S. Felice Circeo | Grass and herbs | - | May 2005 | 1 | | + | - | ITA11 | *ftsZ* |
| T1 | *T. urticae* | France | Vireux | Blackthorn | *Prunus spinosa* | July 2006 | 1 | | - | + | | |
| T2 | *T. urticae* | Unknown | Unknown | Cucumber* | *Cucumis sativus* | * | 1 | | + | - | | |
| T3 | *T. urticae* | Spain | Valencia | ? | ? (Lamiaceae) | May 2004 | 1 | | + | - | | |
| CH1 | *P. harti* | China | Huanyaguan | Soursob | *Oxalis pes-caprae* | Aug 2005 | 1 | | - | + | | |

Listed are sample population code, host species name, sample location (country and locality), host plant, and collection date. Each entry represents a different site of collection (population). More than one individual at a site was examined in some cases (n). ‘W’ and ‘C’ indicate whether *Wolbachia* and/or *Cardinium* were detected (+ = detected; - = not detected). If the analyzed individuals within a population differ for infection status, these are listed separately (e.g., PL5_1 and PL5_2). NL3_9-10 means individual NL3_9 and NL3_10. ‘Cloned’ indicates which gene was cloned and subsequently sequenced for which sample. * = maintained in the lab on bean (*Phaseolus vulgaris*) for over 10 years.
