# Supplementary material for: Diversity and recombination in Wolbachia and Cardinium from Bryobia spider mites
Source: BMC Microbiol. 2012 Jan 18;12(Suppl 1):S13. doi: 10.1186/1471-2180-12-S1-S13 (PMC3287510; doi:10.1186/1471-2180-12-S1-S13)
Supplement: Additional file 2 — Allelic profiles for each of the 37 unique Wolbachia STs. [file 1471-2180-12-S1-S13-S2.pdf]

Additional file 2 - Allelic profiles for each of the 37 unique *Wolbachia* STs

ST	*wsp*	*ftsZ*	*groEl*	*trmD*	Freq.	Clonal complex	Species	Samples
1	1	1	4	1	1	I	BK-B	GR2
2	1	2	4	1	12	I	BK-B	BEL2, ITA5, NL3_1-8, NL4, SA1
3	5	2	4	18	1	-	BspI	NL14
4	1	2	8	1	2	I	BK-B	FR2_1, NL6_2
5	1	2	8	10	1	I	BK-B	NL6_1
6	5	2	8	6	1	I	BK-D	SP3_2
7	12	2	8	8	1	-	BK-D	SP5
8	1	2	9	7	1	-	BK-D	SP1
9	1	2	8	3	1	I	BK-B	FR2_2
10	5	3	8	15	1	III	BspI	BEL4_1
11	1	2	8	6	1	I	BK-D	SP3_3
12	4	3	8	15	1	III	BR	FR14_1
13	5	3	8	5	1	III	BR	FR15
14	5	3	4	1	1	III	BK-D	NL3_9
15	3	3	8	1	1	III	BK-D	NL1_1
16	5	3	8	1	2	III	BK-D	NL1_2, NL3_10
17	7	4	4	16	1	-	BK-A	NL9
18	8	5	1	13	1	-	TU	T2
19	4	6	5	15	1	-	BR	PL5_2
20	5	7	6	17	4	-	BR	NL16_1-4
21	4	8	10	15	1	-	BK-A	FR13
22	5	8	4	15	1	-	BP	NL12
23	9	9	2	12	1	-	TU	T3
24	12	10	12	8	3	V	BK-D	POR1, SP2, SP3_1
25	12	10	11	8	1	V	BK-D	SP4_1
26	13	10	4	1	1	-	BK-B	BEL1_1
27	12	10	8	2	1	II	BK-B	NL7
28	12	10	8	6	1	II	BK-D	SP4_2
29	12	10	8	14	1	II	BspI	BEL4_2
30	12	10	8	1	1	II	BK-B	BEL1_2
31	10	11	4	4	4	-	BR	NL15_1-4
32	11	13	3	9	1	-	BB	FR17
33	6	14	3	9	8	IV	BS	BEL5, FR16_1-5, FR21_1, FR21_3
34	6	14	3	11	1	IV	BS	BEL6
35	-	15	13	19	1	-	BspV	ITA11
36	5	3	8	17	1	III	BR	PL5_1
37	2	12	4	15	1	-	BK-C	US1

Indicated is how many times each ST was found (Freq.), to which clonal complex each ST belongs, in which species it was detected (for abbreviations see legend Figure 2), and in which individual sample (for codes see Additional file 1).
