# Supplementary material for: Diversity and recombination in Wolbachia and Cardinium from Bryobia spider mites
Source: BMC Microbiol. 2012 Jan 18;12(Suppl 1):S13. doi: 10.1186/1471-2180-12-S1-S13 (PMC3287510; doi:10.1186/1471-2180-12-S1-S13)
Supplement: Additional file 3 — Wolbachia gene phylogenies (wsp, ftsZ, groEL, and trmD). [file 1471-2180-12-S1-S13-S3.pdf]

Additional file 3 - *Wolbachia* gene phylogenies (*wsp*, *ftsZ*, *groEL*, and *trmD*)

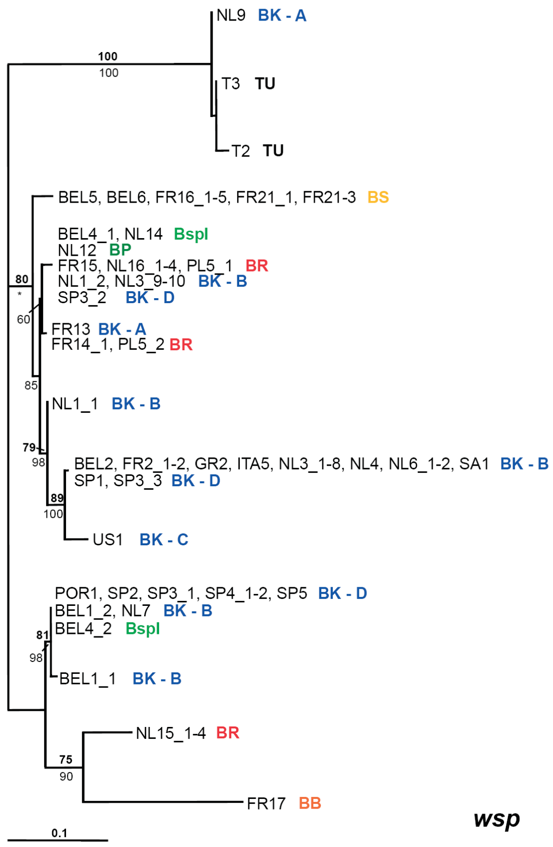

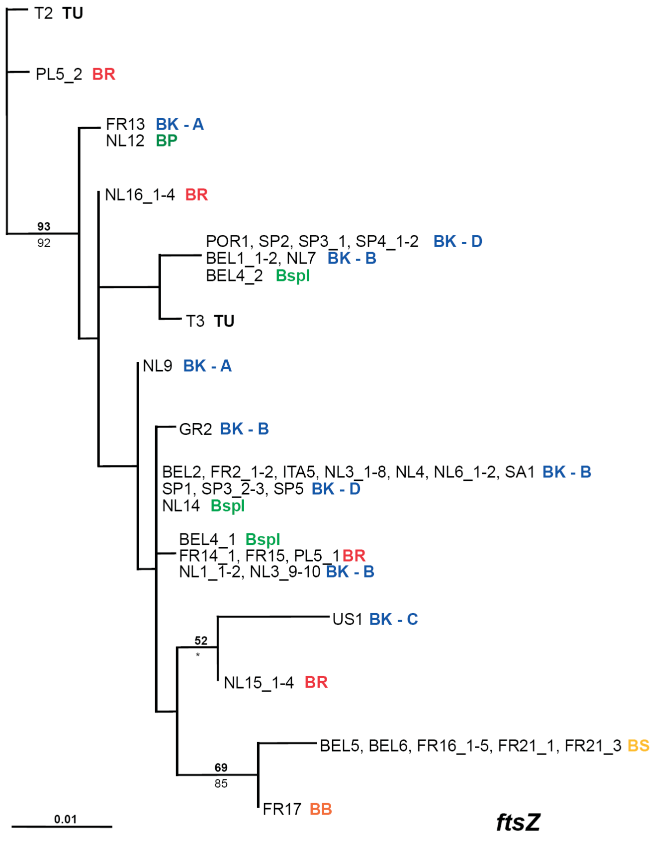

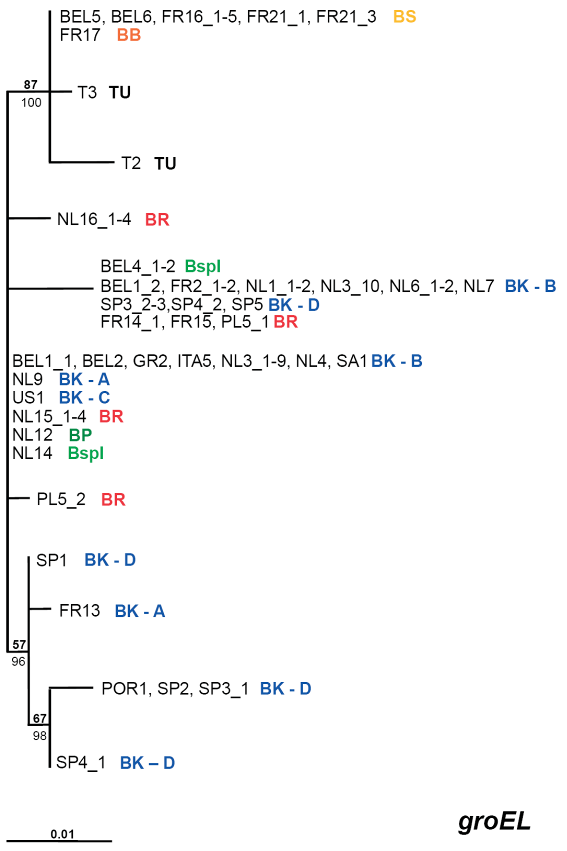

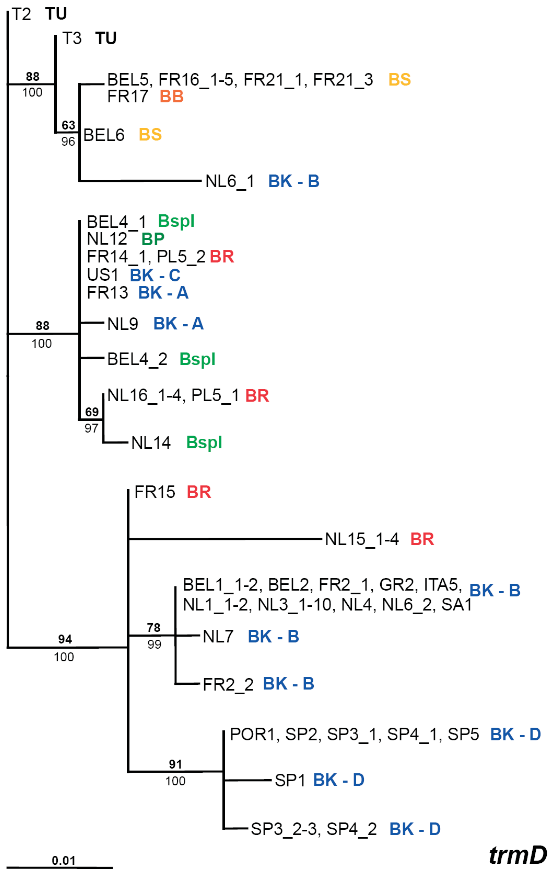

Sample code (Additional file 1) and host species name in which each strain was detected are indicated (for abbreviations see legend Figure 2). * = the topology within this clade is slightly different for the MrBayes topology. ML bootstrap values (top number, bold) and Bayesian posterior probabilities (bottom number, plain) are depicted (only values larger than 50 are indicated). The bar at the bottom indicates a branch length of 1% (*ftsZ*, *groEL*, and *trmD*) or 10% (wsp) likelihood distance.
