# Supplementary material for: Diversity and recombination in Wolbachia and Cardinium from Bryobia spider mites
Source: BMC Microbiol. 2012 Jan 18;12(Suppl 1):S13. doi: 10.1186/1471-2180-12-S1-S13 (PMC3287510; doi:10.1186/1471-2180-12-S1-S13)
Supplement: Additional file 4 — GenBank accession numbers. [file 1471-2180-12-S1-S13-S4.pdf]

Additional file 4 – GenBank accession numbers

Code	Species[a]	Country	Genbank accession number					
			wsp	ftsZ	groEL	trmD	16S rDNA	gyrB
BEL1_1	*B. kissophila*	Belgium	***JN572868***	JN572854	JN572836	JN572828	n.a.	n.a.
BEL1_2	*B. kissophila*	Belgium	JN572866	JN572854	***JN572841***	***JN572828***	n.a.	n.a.
BEL2	*B. kissophila*	Belgium	***JN572875***	***JN572850***	***JN572836***	JN572828	n.a.	n.a.
FR2_1	*B. kissophila*	France	JN572875	JN572850	JN572841	JN572828	n.a.	n.a.
FR2_2	*B. kissophila*	France	JN572875	JN572850	JN572841	***JN572829***	n.a.	n.a.
FR13	*B. kissophila*	France	***JN572873***	***JN572863***	***JN572846***	JN572821	n.a.	n.a.
GR2	*B. kissophila*	Greece	JN572875	***JN572849***	JN572836	JN572828	n.a.	n.a.
ITA5	*B. kissophila*	Italy	JN572875	JN572850	JN572836	JN572828	n.a.	n.a.
NL1_1	*B. kissophila*	Netherlands	***JN572877***	***JN572853***	JN572841	JN572828	n.a.	n.a.
NL1_2	*B. kissophila*	Netherlands	***JN572872***	JN572853	JN572841	JN572828	n.a.	n.a.
NL3_1	*B. kissophila*	Netherlands	JN572875	JN572850	JN572836	JN572828	n.a.	n.a.
NL3_2	*B. kissophila*	Netherlands	JN572875	JN572850	JN572836	JN572828	n.a.	n.a.
NL3_3	*B. kissophila*	Netherlands	JN572875	JN572850	JN572836	JN572828	n.a.	n.a.
NL3_4	*B. kissophila*	Netherlands	JN572875	JN572850	JN572836	JN572828	n.a.	n.a.
NL3_5	*B. kissophila*	Netherlands	JN572875	JN572850	JN572836	JN572828	n.a.	n.a.
NL3_6	*B. kissophila*	Netherlands	JN572875	JN572850	JN572836	JN572828	n.a.	n.a.
NL3_7	*B. kissophila*	Netherlands	JN572875	JN572850	JN572836	JN572828	n.a.	n.a.
NL3_8	*B. kissophila*	Netherlands	JN572875	JN572850	JN572836	JN572828	n.a.	n.a.
NL3_9	*B. kissophila*	Netherlands	JN572872	JN572853	JN572836	JN572828	n.a.	n.a.
NL3_10	*B. kissophila*	Netherlands	JN572872	JN572853	JN572841	JN572828	n.a.	n.a.
NL4	*B. kissophila*	Netherlands	JN572875	JN572850	JN572836	JN572828	n.a.	n.a.
NL6_1	*B. kissophila*	Netherlands	JN572875	JN572850	JN572841	***JN572814***	n.a.	n.a.
NL6_2	*B. kissophila*	Netherlands	JN572875	JN572850	JN572841	JN572828	n.a.	n.a.
NL7	*B. kissophila*	Netherlands	JN572866	JN572854	JN572841	***JN572830***	n.a.	n.a.
NL9	*B. kissophila*	Netherlands	***JN572881***	***JN572861***	JN572836	***JN572817***	n.a.	n.a.
POR1	*B. kissophila*	Portugal	***JN572866***	***JN572854***	***JN572843***	***JN572825***	n.a.	n.a.
SA1	*B. kissophila*	South Africa	JN572875	JN572850	JN572836	JN572828	n.a.	n.a.
SP1	*B. kissophila*	Spain	JN572875	JN572850	***JN572845***	***JN572827***	n.a.	n.a.
SP2	*B. kissophila*	Spain	JN572866	JN572854	JN572843	JN572825	n.a.	n.a.
SP3_1	*B. kissophila*	Spain	JN572866	JN572854	JN572843	JN572825	n.a.	n.a.
SP3_2	*B. kissophila*	Spain	JN572872	JN572850	JN572841	***JN572826***	n.a.	n.a.
SP3_3	*B. kissophila*	Spain	JN572875	JN572850	JN572841	JN572826	n.a.	n.a.
SP4_1	*B. kissophila*	Spain	JN572866	JN572854	***JN572844***	JN572825	n.a.	n.a.
SP4_2	*B. kissophila*	Spain	JN572866	JN572854	JN572841	JN572826	n.a.	n.a.
SP5	*B. kissophila*	Spain	JN572866	JN572850	JN572841	JN572825	n.a.	n.a.
US1	*B. kissophila*	United Sates	***JN572876***	***JN572857***	JN572836	***JN572821***	n.a.	n.a.
NL12	*B. praetiosa*	Netherlands	***JN572870***	EU499322	EU499332	***JN572819***	n.a.	n.a.

Code	Species[a]	Country	Genbank accession number					
			wsp	ftsZ	groEL	trmD	16S rDNA	gyrB
FR14	*B. rubrioculus*	France	***JN572874***	***JN572852***	***JN572842***	***JN572820***	JN572883	***JN572808***
FR15	*B. rubrioculus*	France	***JN572871***	JN572852	JN572842	***JN572831***	JN572883	***JN572809***
NL15_1	*B. rubrioculus*	Netherlands	***JN572864***	***JN572860***	***JN572837***	***JN572832***	***JN572883***	***JN572805***
NL15_2	*B. rubrioculus*	Netherlands	JN572864	JN572860	JN572837	JN572832	JN572883	JN572805
NL15_3	*B. rubrioculus*	Netherlands	JN572864	JN572860	JN572837	JN572832	JN572883	JN572805
NL15_4	*B. rubrioculus*	Netherlands	JN572864	JN572860	JN572837	JN572832	JN572883	JN572805
NL16_1	*B. rubrioculus*	Netherlands	JN572871	***JN572862***	***JN572839***	***JN572822***	n.a.	n.a.
NL16_2	*B. rubrioculus*	Netherlands	JN572871	JN572862	JN572839	JN572822	n.a.	n.a.
NL16_3	*B. rubrioculus*	Netherlands	JN572871	JN572862	JN572839	JN572822	n.a.	n.a.
NL16_4	*B. rubrioculus*	Netherlands	JN572871	JN572862	JN572839	JN572822	n.a.	n.a.
PL5_1	*B. rubrioculus*	Poland	JN572871	JN572852	JN572842	JN572822	***JN572887***	***JN572804***
PL5_2	*B. rubrioculus*	Poland	JN572874	***JN572848***	***JN572838***	JN572820	n.a.	n.a.
BEL5	*B. sarothamni*	Belgium	***JN572878***	***JN572858***	***JN572834***	***JN572811***	n.a.	n.a.
BEL6	*B. sarothamni*	Belgium	JN572878	JN572858	JN572834	***JN572813***	n.a.	n.a.
FR16_1	*B. sarothamni*	France	JN572878	EU499320	EU499330	JN572811	n.a.	n.a.
FR16_2	*B. sarothamni*	France	JN572878	EU499320	EU499330	JN572811	***JN572886***	***JN572802***
FR16_3	*B. sarothamni*	France	JN572878	EU499320	EU499330	JN572811	n.a.	n.a.
FR16_4	*B. sarothamni*	France	JN572878	EU499320	EU499330	JN572811	JN572886	JN572802
FR16_5	*B. sarothamni*	France	JN572878	EU499320	EU499330	JN572811	JN572886	JN572802
FR21_1	*B. sarothamni*	France	JN572878	JN572858	JN572834	JN572811	***JN572888***	***JN572803***
FR21_2	*B. sarothamni*	France	n.a.	n.a.	n.a.	n.a.	JN572888	JN572803
FR21_3	*B. sarothamni*	France	JN572878	JN572858	JN572834	JN572811	***JN572884***	***JN572806***
FR17	*B. berlesei*	France	***JN572865***	***JN572859***	***JN572835***	***JN572812***	n.a.	n.a.
BEL4_1	*B.*spec. I	Belgium	***JN572869***	***JN572851***	***JN572840***	***JN572818***	n.a.	n.a.
BEL4_2	*B.*spec. I	Belgium	***JN572867***	***JN572855***	JN572840	***JN572824***	n.a.	n.a.
NL14	*B.*spec. I	Netherlands	JN572869	EU499323	EU499333	***JN572823***	n.a.	n.a.
ITA11	*B.*spec. V	Italy	n.a.	EU499321	EU499331	***JN572833***	n.a.	n.a.
T1	*T. urticae*	France	n.a.	n.a.	n.a.	n.a.	***JN572882***	***JN572807***
T2	*T. urticae*	Unknown	***JN572880***	EU499324	EU499334	***JN572816***	n.a.	n.a.
T3	*T. urticae*	Spain	***JN572879***	***JN572856***	***JN572847***	***JN572815***	n.a.	n.a.
CH1	*P. harti*	China	n.a.	n.a.	n.a.	n.a.	***JN572885***	***JN572810***

GenBank accession numbers for all sequences used in this study. Identical haplotypes within a host species (see Figure 5 (*Cardinium*) and Additonal file 3 (*Wolbachia*)) have identical accession numbers. Numbers newly submitted to GenBank are depicted in italics.
